# Supplementary figures and images for: Computational modeling of ketamine-induced changes in gamma-band oscillations: The contribution of parvalbumin and somatostatin interneurons
Source: PLoS Comput Biol. 2025 Jun 9;21(6):e1013118. doi: 10.1371/journal.pcbi.1013118 (PMC12204622; doi:10.1371/journal.pcbi.1013118)

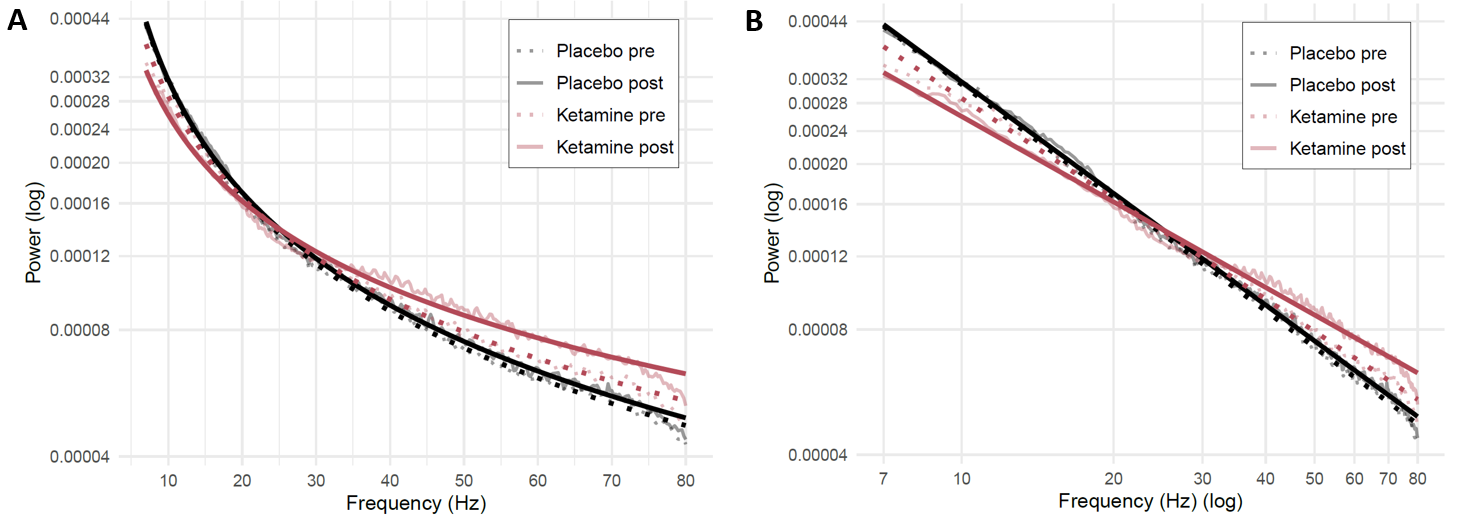

Supplement: S1 Fig — (A) Power log-transformed. (B) Power and Frequency log-transformed. (PNG) [file pcbi.1013118.s001.png]

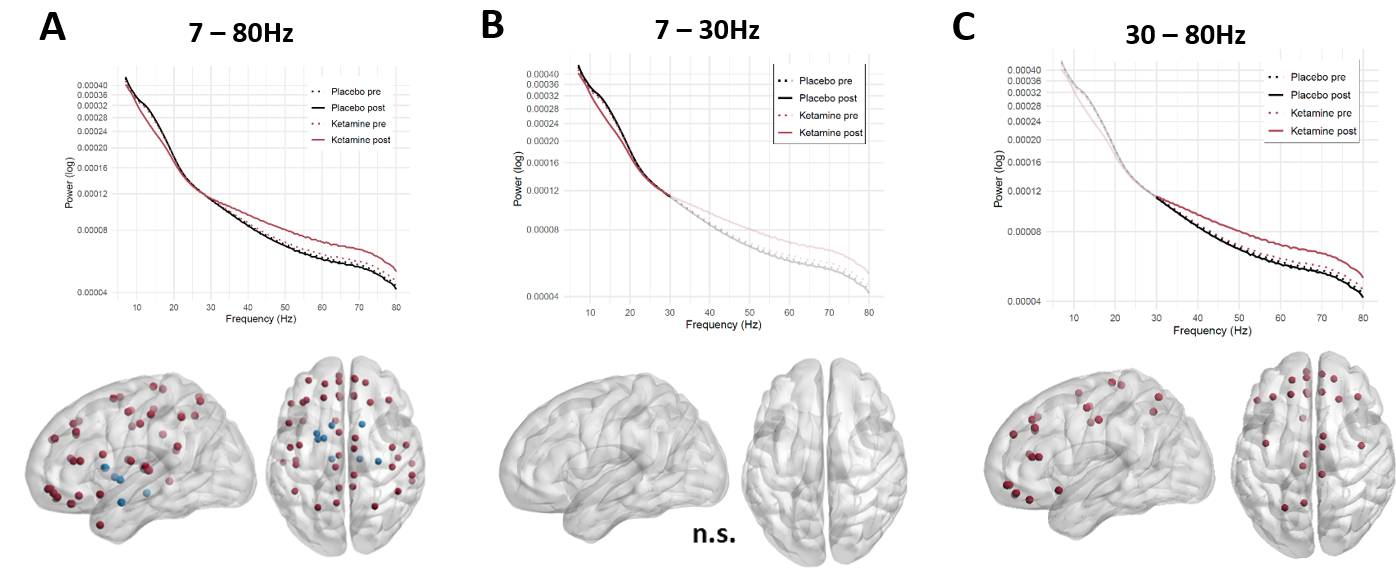

Supplement: S2 Fig — (A) Upper panel: Power spectrum (log-transformed) of the aperiodic component per condition, averaged across regions with significant slope change and across participants, in a frequency range between 7 and 80 Hz. Lower panel: Centroids of cortical (red) and subcortical (blue) brain regions with significant slope change (slope fitted between 7 and 80 Hz). Perspective from the left and above on a semi-transparent brain. (B-C) Same as (A) but in a frequency range of 7–30 Hz (B) and 30–80 Hz (C). (PNG) [file pcbi.1013118.s002.png]

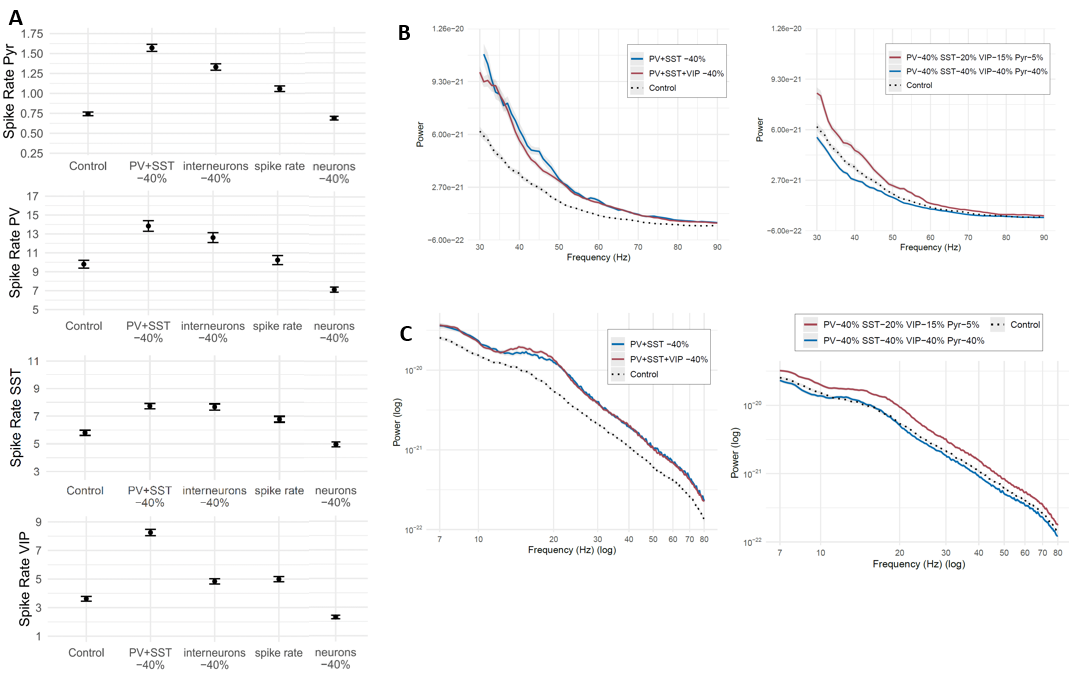

Supplement: S3 Fig — (A) Error bars indicate standard error of the mean. Control condition without any manipulations. In the test conditions, NMDA receptors of different combinations of parvalbumin neurons (PV), somatostatin neurons (SST), pyramidal neurons (Pyr) and vasoactive-intestinal peptide neurons (VIP) were reduced by the indicated amount. In the ‘spike rate’ condition, NMDA receptors were reduced proportional to the spike rate of that neuron type (NMDA receptor reductions: PV -40%, SST -20%, VIP -15%, Pyr -5%). In the ‘interneurons’ condition, NMDA receptors of PV, SST, and VIP interneurons were reduced by 40%. In the ‘neurons’ condition, NMDA receptors of all modeled neurons types (PV, SST, VIP, and pyramidal neurons) were reduced by 40%. (B) Averaged power spectrum of the fast-Fourier transformed data in the gamma-power range. (C) Aperiodic component of the power spectrum in log-log space. Shaded envelopes indicate standard error. Control condition (black, dotted line) without any manipulations. In the test conditions (straight, colored lines), NMDA receptors of PV, SST, VIP, and Pyr neurons, were reduced by the indicated amount. (PNG) [file pcbi.1013118.s003.png]
