# Supplementary material for: Computational modeling of ketamine-induced changes in gamma-band oscillations: The contribution of parvalbumin and somatostatin interneurons
Source: PLoS Comput Biol. 2025 Jun 9;21(6):e1013118. doi: 10.1371/journal.pcbi.1013118 (PMC12204622; doi:10.1371/journal.pcbi.1013118)
Supplement: S1 File — (DOCX) [file pcbi.1013118.s004.docx]

**S1 File: AAL region abbreviations**

| **PreCG** | Precentral gyrus | **LING** | Lingual gyrus |
| --- | --- | --- | --- |
| **SFGdor** | Superior frontal gyrus (dorsolateral) | **SOG** | Superior occipital gyrus |
| **ORBsup** | Superior frontal gyrus (orbital) | **MOG** | Middle occipital gyrus |
| **MFG** | Middle frontal gyrus | **IOG** | Inferior occipital gyrus |
| **ORBmid** | Middle frontal gyrus (orbital) | **FFG** | Fusiform gyrus |
| **IFGoperc** | Inferior frontal gyrus (opercular) | **PoCG** | Postcentral gyrus |
| **IFGtriang** | Inferior frontal gyrus (triangular) | **SPG** | Superior parietal gyrus |
| **ORBinf** | Inferior frontal gyrus (orbital) | **IPG** | Inferior parietal gyrus |
| **ROL** | Rolandic operculum | **SMG** | Supramarginal gyrus |
| **SMA** | Supplementary motor area | **ANG** | Angular gyrus |
| **OLF** | Olfactory cortex | **PCUN** | Precuneus |
| **SFGmed** | Superior frontal gyrus (medial) | **PCL** | Paracentral lobule |
| **ORBsupmed** | Superior frontal gyrus (medial orbital) | **CAU** | Caudate |
| **REC** | Rectus gyrus | **PUT** | Putamen |
| **INS** | Insula | **PAL** | Pallidum |
| **ACG** | Anterior cingulate gyrus | **THA** | Thalamus |
| **MCG** | Medial cingulate gyrus | **HES** | Heschl gyrus |
| **PCG** | Posterior cingulate gyrus | **STG** | Superior temporal gyrus |
| **HIP** | Hippocampus | **TPOsup** | Temporal pole (superior) |
| **PHG** | Parahippocampal gyrus | **MTG** | Middle temporal gyrus |
| **AMYG** | Amygdala | **TPOmid** | Temporal pole (middle) |
| **CAL** | Calcarine cortex | **ITG** | Inferior temporal gyrus |
| **CUN** | Cuneus |  |  |
