# Supplementary material for: Computational modeling of ketamine-induced changes in gamma-band oscillations: The contribution of parvalbumin and somatostatin interneurons
Source: PLoS Comput Biol. 2025 Jun 9;21(6):e1013118. doi: 10.1371/journal.pcbi.1013118 (PMC12204622; doi:10.1371/journal.pcbi.1013118)
Supplement: S2 File — (DOCX) [file pcbi.1013118.s005.docx]

**S2 File: Power Differences in Different Frequency Ranges**

**Human MEG Data**

As additional analysis, we analyzed Ketamine-induced power changes in the theta (4-8 Hz), alpha (8-12 Hz), and beta (13-30 Hz) frequency ranges in both the non-separated as well as the periodic power spectrum. Even though a trend of increased theta power and decreased alpha power can be seen (Fig S2.1), which is in line with results of former research [1-3], statistical testing (FDR-corrected t-tests) revealed no significant theta or alpha power changes in any brain region (p > 0.05). However, significant Ketamine-induced decreases in the beta range in several brain regions could be observed, both in the periodic and the non-separated data, confirming our previous findings [4] (Fig S2.2 and Table S2.1).


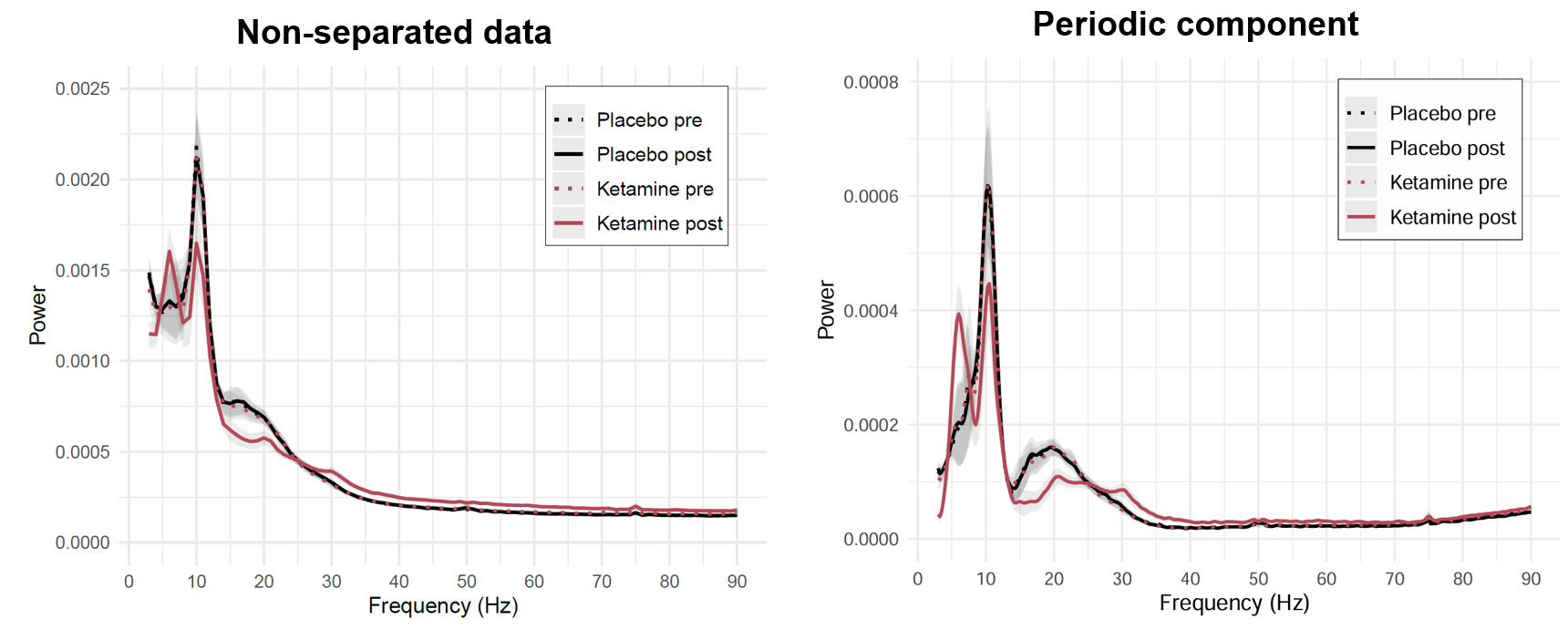


**S2.1 Fig. Power spectra of the non-separated, Fourier-transformed data (left panel) and the periodic component (right panel) of the data.** Hanning-tapered. Averaged across all virtual channel regions. The shaded envelopes indicate the standard error. black: Placebo, red: Ketamine, dotted line: pre infusion onset, straight line: post infusion onset.


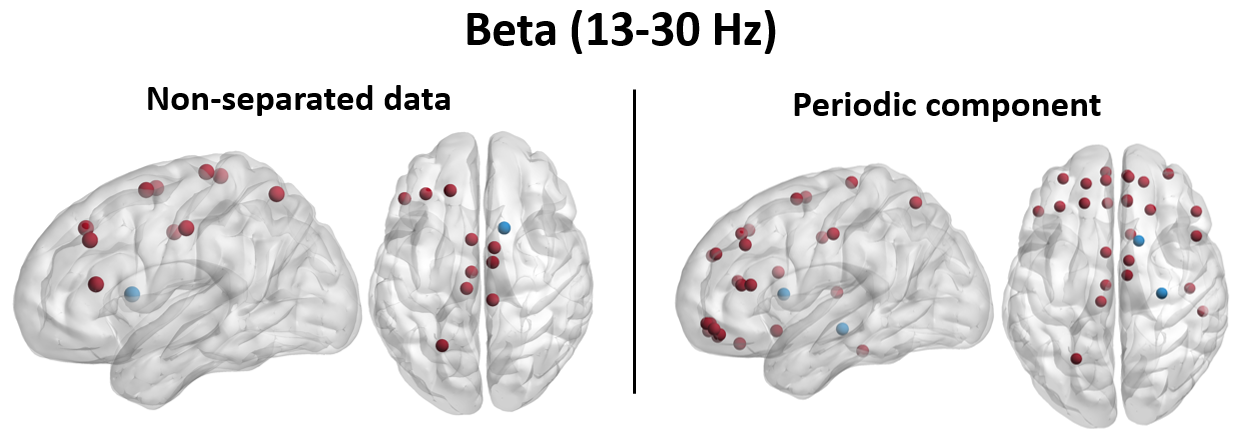


**S2.2 Fig. Centroids of cortical (red) and subcortical (blue) brain regions with significant beta power change.** Perspective from the left and above on a semi-transparent brain. Labels of these regions can be found in Table S2.1.

|  | **Significant brain regions** | **t-values** | **p-values** |
| --- | --- | --- | --- |
| **Beta difference:**  **non-separated data** |  |  |  |
| Cortical | LSFGdor, LMFG, LIFGtriang, LSMA, RSMA, LDCG, RDCG, LSPG, LPCL, RPCL | 4.025 | 0.001 |
| Subcortical | RCAU | 4.025 | 0.001 |
| **Beta difference:**  **periodic component** |  |  |  |
| Cortical | LSFGdor, RSFGdor, LORBsup, RORBsup, LMFG, LORBmid, RORBmid, RIFGoperc, LIFGtriang, RIFGtriang, LSMA, RSMA, ROLF, LSFGmed, RSFGmed, LORBsupmed, RORBsupmed, RREC, LACG, RACG, LDCG, RDCG, LSPG, LPCL, RHES, RITG | 3.394 to 4.025 | 0.003 to 0.001 |
| Subcortical | RHIP, RCAU | 4.025 | 0.001 |

**S2.1 Table.** **Overview of brain regions with significant Ketamine-induced increase in beta power.** Abbreviations of brain regions according to AAL-atlas, for full labels see S1 File. Prefix: L = left, R = right.

The analysis of gamma-band power in the periodic component revealed a similar picture as in the non-separated data, with significant power increases across a range of cortical and subcortical brain regions (see Fig S2.3), highlighting that oscillatory activity is involved in the Ketamine-induced increases of gamma-band power.


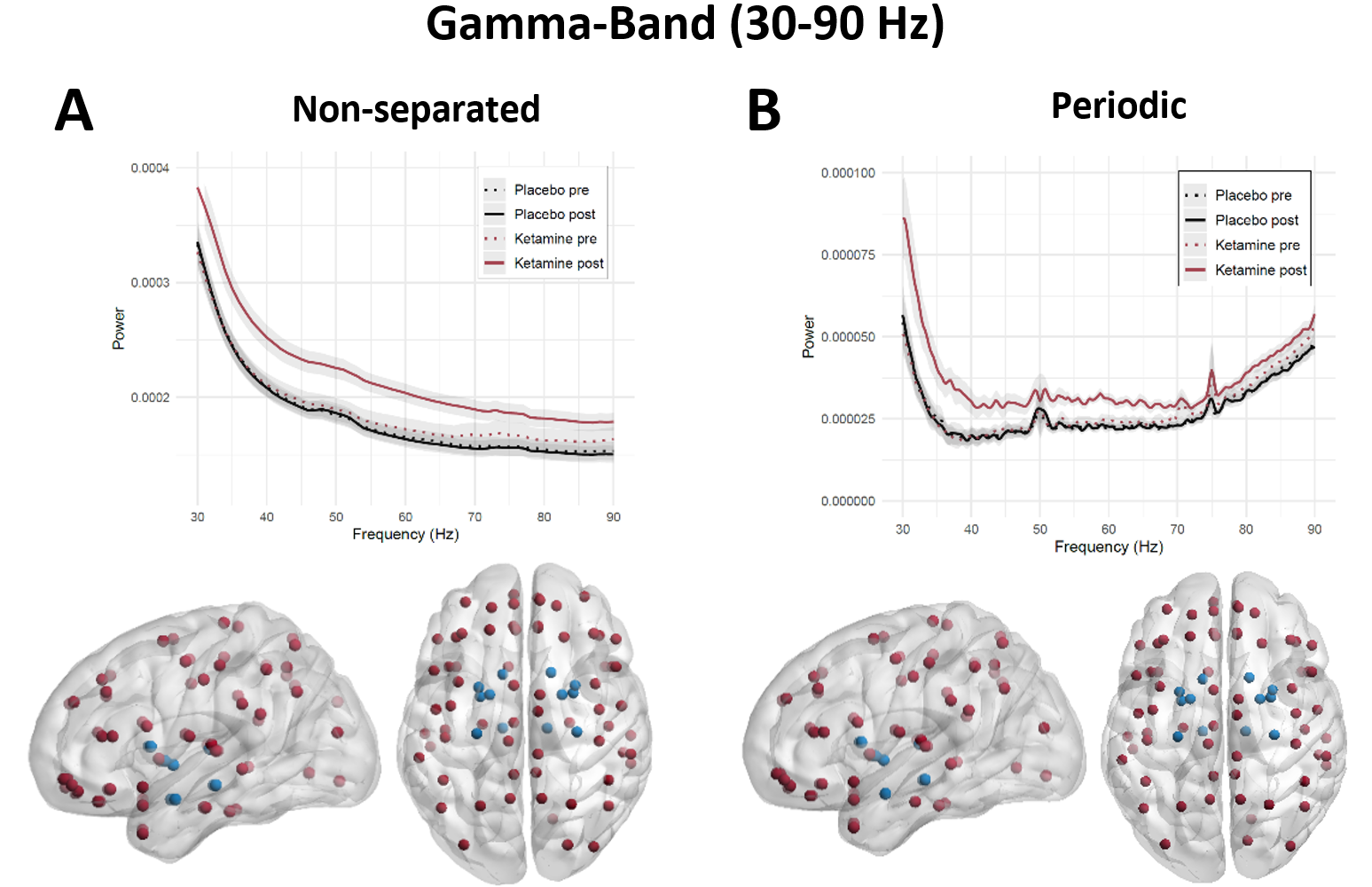


**S2.3 Fig Ketamine-induced changes in gamma-band power in the non-separated (A) and periodic component data (B).** Grand-averaged (n=12) power spectrum in gamma range (30-90 Hz) per condition (black: Placebo, red: Ketamine, dotted line: pre infusion onset, straight line: post infusion onset), averaged across regions with significant gamma-band power change and across participants. Shaded envelope indicates standard error of the mean. Lower panels: Centroids of cortical (red) and subcortical (blue) brain regions with significant gamma-band power change. Perspective from the left and above on a semi-transparent brain.

**Simulated NMDA-R Reductions**

We repeated these analyses with the simulated data (see Fig S2.4 and Table S2.2). Interestingly, a strong increase in alpha power and decrease in theta power could be observed in several conditions, contrasting the results seen in the human MEG data. A decrease in beta power could only be observed in conditions with manipulated NMDA-Rs of pyramidal neurons in the non-separated data; in PV and SST conditions an increase in beta power was found. The results of gamma-band power in the periodic signal confirm largely what was observed in the non-separated data, with increases occurring only in PV conditions (PV -60%) and SST conditions.


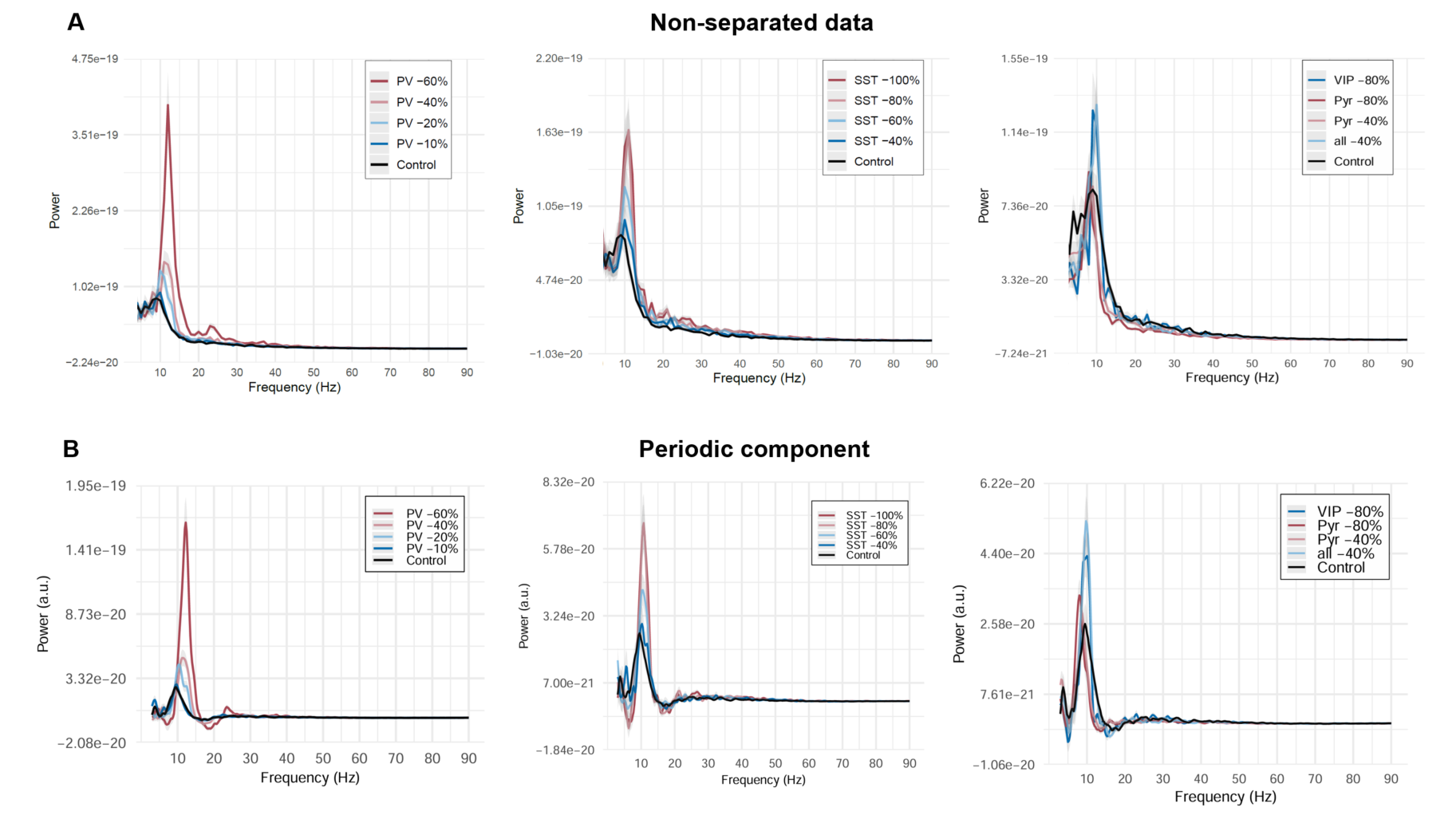


**S2.4 Fig. Power spectra of simulated data with NMDA-R reductions in the non-separated data (A) and periodic component data (B).** Hanning-tapered. The shaded envelopes indicate the standard error. Control condition (black line) without any manipulations. In the test conditions (colored lines), NMDA receptors of parvalbumin interneurons, PV, somatostatin interneurons, SST, vasoactive-intestinal peptide interneurons, VIP, and pyramidal neurons, Pyr, and in all aforementioned neuron types simultaneously, were reduced by the indicated amount.

|  | **Significant simulation conditions (after FDR correction)** | |
| --- | --- | --- |
|  | **non-separated data** | **periodic component** |
| theta (4-8 Hz) | *decrease:*  all Pyr, VIP -80%, all neurons | *decrease:*  PV -60%, all SST (except -40%), VIP -80% |
| alpha (8-12 Hz) | *increase:*  all PV (except -10%), all SST, VIP -80% | *increase:*  all PV (except -10%),  all SST (except -­40%), VIP -80% |
| beta (13-30 Hz) | *increase:*  all PV (except -10%), all SST  *decrease:*  all Pyr | *increase:*  PV -40%, PV -60% |
| beta (15-30 Hz) | *increase:*  all PV (except -10%), all SST  *decrease:*  all Pyr | - |
| gamma (30-90 Hz) | *increase:*  all PV (except -10%), all SST  *decrease:*  all Pyr, all neurons | *increase:*  PV -60%, all SST  *decrease:*  all Pyr, all neurons |

**S2.2 Table. Overview of simulation conditions with significant power changes in different frequency ranges compared to a model without NMDA-R manipulations**. In the simulated conditions, NMDA receptors of parvalbumin interneurons, PV, somatostatin interneurons, SST, vasoactive-intestinal peptide interneurons, VIP, and pyramidal neurons, Pyr, and in all aforementioned neuron types simultaneously, were reduced by the indicated amount.

A potential explanation for the difference in the power spectral changes in the lower frequencies in human data after Ketamine administration vs. simulated data is that the computational layer-23 model targeted only E/I-mechanisms (i.e., NMDA-R output of pyramidal neurons and interneurons). While gamma-band changes may be captured by cortical layer 2/3 pyramidal neuron–interneuron mechanisms, other oscillation frequencies might be more reliant on other neurotransmitter systems, such as cholinergic neurotransmission for alpha and beta [5] and theta [6] , as well as on additional brain structures, such as hippocampus for theta [7], or other cortical layers (e.g. deeper layers for alpha [8]).

**References**

1. Curic S, Andreou C, Nolte G, Steinmann S, Thiebes S, Polomac N, et al. Ketamine alters functional gamma and theta resting-state connectivity in healthy humans: Implications for schizophrenia treatment targeting the glutamate system. Frontiers in psychiatry. 2021;12:671007.

2. De la Salle S, Choueiry J, Shah D, Bowers H, McIntosh J, Ilivitsky V, et al. Effects of ketamine on resting-state EEG activity and their relationship to perceptual/dissociative symptoms in healthy humans. Frontiers in Pharmacology. 2016;7:348.

3. Muthukumaraswamy SD, Shaw AD, Jackson LE, Hall J, Moran R, Saxena N. Evidence that subanesthetic doses of ketamine cause sustained disruptions of NMDA and AMPA-mediated frontoparietal connectivity in humans. Journal of Neuroscience. 2015;35(33):11694-706.

4. Rivolta D, Heidegger T, Scheller B, Sauer A, Schaum M, Birkner K, et al. Ketamine dysregulates the amplitude and connectivity of high-frequency oscillations in cortical–subcortical networks in humans: evidence from resting-state magnetoencephalography-recordings. Schizophrenia bulletin. 2015;41(5):1105-14.

5. Bauer M, Kluge C, Bach D, Bradbury D, Heinze HJ, Dolan RJ, et al. Cholinergic enhancement of visual attention and neural oscillations in the human brain. Current Biology. 2012;22(5):397-402.

6. Liljenstrom H, Hasselmo ME. Cholinergic modulation of cortical oscillatory dynamics. Journal of Neurophysiology. 1995;74(1):288-97.

7. Pignatelli M, Beyeler A, Leinekugel X. Neural circuits underlying the generation of theta oscillations. Journal of Physiology-Paris. 2012;106(3-4):81-92.

8. Bollimunta A, Chen Y, Schroeder CE, Ding M. Neuronal mechanisms of cortical alpha oscillations in awake-behaving macaques. Journal of Neuroscience. 2008;28(40):9976-88.
