# Supplementary material for: Computational modeling of ketamine-induced changes in gamma-band oscillations: The contribution of parvalbumin and somatostatin interneurons
Source: PLoS Comput Biol. 2025 Jun 9;21(6):e1013118. doi: 10.1371/journal.pcbi.1013118 (PMC12204622; doi:10.1371/journal.pcbi.1013118)
Supplement: S3 File — (DOCX) [file pcbi.1013118.s006.docx]

**S3 File: Statistical information on computational scenarios after reduction of NMDA-Rs in combinations of neurons**

Simulated conditions with reductions in both PV+ and SST+ neurons (*p* < 0.001, *t* = -16.00), all interneurons (*p* < 0.001, *t* = -13.42), and reductions according to spike rate (PV -40%, SST -20%, PV -15%, pyramidal neurons -5%; *p* < 0.001, *t* = -8.15), showed enhanced gamma-band power. In contrast, comparable reductions in all neuron types did not show significantly higher gamma-band power.
