# Supplementary material for: Computational modeling of ketamine-induced changes in gamma-band oscillations: The contribution of parvalbumin and somatostatin interneurons
Source: PLoS Comput Biol. 2025 Jun 9;21(6):e1013118. doi: 10.1371/journal.pcbi.1013118 (PMC12204622; doi:10.1371/journal.pcbi.1013118)
Supplement: Attachment — Submitted filename: Response to Reviewers.docx [file pcbi.1013118.s008.docx]

**Response to Reviewers**

**Reviewer #1:**

**This paper employs an impressive range of research modalities to address a central question in neuroscience: how cellular and receptor level phenomena can lead to brain-level behaviors. Significantly, it uses methods that span levels of analysis—tissue level computational modeling; gene profiling of receptor expression across brain regions; and conventional (gamma band) as well as more innovative (aperiodic power spectrum) analyses applied to MEG data—to arrive at its conclusions. The power of this approach is that it can produce non-obvious insights into how these micro-level effects give rise to emergent behaviors. While the N used in this study is relatively small, this is more than made up for by the fact that it is highly innovative methodologically—I feel that this is perhaps the most important contribution of this paper.**

***Response:*** *We thank the reviewer for the positive assessment of our work and the suggested improvements that we have incorporated.*

**1) The authors may wish to highlight the literature on the aperiodic power spectrum, its examination in the human brain and its potential functional significance. Also, an explanation of this should not be buried in the Supplementary Materials but included in the Methods and described in a way that provides some “intuition” for those less technically inclined.**

***Response:*** *We thank* *the reviewer for highlighting this point. In the revised version of the manuscript, we added a more elaborate depiction of the literature on its functional significance:*

*“Moreover, recent evidence suggests that the slope of the aperiodic, non-oscillatory component of brain activity may provide an additional marker for E/I-balance [9], as it has been shown to reflect the integration of excitatory and inhibitory synaptic currents [10] and been associated with working memory performance [9, 11], consciousness [12, 13], and psychiatric disorders, such as depression, attention deficit disorder, and autism [14]”*

*In addition, we agree that the description of the slope calculation is an important detail and have included it in the main text.*

**2) The work in in its current form it comes across as something of a “technical exercise”. It could be improved by a greater discussion of its clear relevance to a clinical problem. A greater development of the potential relevance to Schizophrenia patients (SZ) (e.g., how this work may shed light on an underlying cause of SZ, or illuminate how neuropathology may lead to functional deficits, or point the way to better treatments) would improve the paper and make it interesting to a wider audience.**

***Response:*** *We agree that a wider discussion of the relevance for SZ is important. Therefore, we added the following discussion:*

*“The current approach combining MEG-activity with computational modelling to elucidate the mechanisms of E/I-balance alterations is also relevant for understanding schizophrenia, which is characterized by prominent alterations in gamma-band oscillations [64] as well as alterations in excitatory and inhibitory neurotransmission [65]. However, it is currently unclear whether impairments in GABAergic interneurons, for example, are a primary dysfunction or whether these reflect a compensatory deficit towards impaired excitatory inputs [66]. Accordingly, advances in computational modelling could contribute towards disentangling competing circuit hypotheses [67].”*

**Reviewer #2:**

**This work is a nice combination of approaches showing MEG changes in participants in response to ketamine, linking these effects to gene expression, and using computational models to uncover associated underlying microcircuit mechanisms. In general, while I find the work very interesting, I think that the gene expression analyses could benefit from being expanded and the simulations can be refined accordingly to better link with the gene expression findings and strengthen the validity of the simulations. Below I outline this and several other key points for consideration.**

***Response:*** *We thank the reviewer for the supportive assessment of our work.*

**1) If no change is seen in the slope of the aperiodic component in the lower frequency range (Fig S2), it seems like changes in gamma power may be driving the change in the aperiodic slope. So, is the change in gamma power and change in aperiodic slope just two measurements of the same effect? Can the authors demonstrate that the slope changes are not driven by gamma power entirely (e.g. using subtraction methods) to warrant including the aperiodic slope feature? It may also be helpful to see more examples of aperiodic fits in Fig S1 to see a range of aperiodic slopes.**

***Response****: We agree that it is critical that changes in gamma-band power and the aperiodic slope are not merely two measurements of the same underlying effect.*

*We chose both measures as estimates for E/I-balance and therefore also expected them to be correlated, while showing slightly different aspects of E/I-balance (see Ahmad et al., 2022 for a review).*

*Moreover, a power increase at higher frequencies (30-90 Hz), at least in the aperiodic component, can be regarded as a mere consequence of a flatter slope, as long as the aperiodic offset is not considerably different between conditions as in our data. Indeed, using Pearson correlations across subjects, we observed a high, significant correlation between slope and aperiodic gamma-band power (rho = -.86; p < .001). This relationship, however, is not perfect, indicating that they do not fully overlap. While both measures are highly correlated, it is not possible to disentangle this effect and to show that changes in gamma-band power are not driving the change in aperiodic slope.*

*While acknowledging that the two measures are closely related, the two measures are not identical, and we believe that they do provide different insights into the effects of Ketamine on E/I-balance.*

**2) From the plots showing the power spectra in the aperiodic range, there does appear as though there may be ketamine-associated decreases in lower frequency peaks. Can the authors do a more complete power spectral decomposition of the periodic component (for both the MEG data and the simulated MEG) to quantify if there are changes in theta, alpha, beta, and gamma peaks after the aperiodic component is subtracted?**

***Response:*** *We agree that the analysis of other frequency bands after ketamine administration might yield interesting results. Our rational not to include these analyses in our manuscript was our focus on potential E/I-related measures, of which gamma-band power and aperiodic exponent are the most promising candidates.*

*An analysis of beta-band power after ketamine administration from our dataset has also already been published by Rivolta et al. (2015), although their MEG analysis and source reconstruction pipeline was different. Nevertheless, we agree that looking at power differences in different frequency bands after subtracting the aperiodic component might reveal new insights and is therefore a valuable suggestion.*

*In the following, the power spectra of both the periodic component and, for comparison, the typical, non-separated fourier-transformed data are shown (hanning-tapered; averaged across all virtual channel regions; the shaded envelopes indicate the standard error).*

*As can be seen, lower frequency ranges, including theta (4-8 Hz) and alpha (8-12 Hz) band, are characterized by high variability, in both non-separated and periodic data. Even though a trend of increased theta power and decreased alpha power can be seen, which is in line with results from other groups (Muthukumaraswamy et al., 2015; De la Salle et al., 2016; Curic et al., 2021) statistical testing at the virtual channel level (FDR-corrected t-tests) revealed no significant theta or alpha power changes in any brain region (p > 0.05).*

*However, significant ketamine-induced decreases in the beta (13-30 Hz) range in several brain regions could be observed, both in the periodic and the non-separated data, confirming our previous findings (Rivolta et al., 2015).*

|  | **Significant brain regions** | **t-values** | **p-values** |
| --- | --- | --- | --- |
| **Beta difference:**  **non-separated data** |  |  |  |
| *Cortical* | LSFGdor, LMFG, LIFGtriang, LSMA, RSMA, LDCG, RDCG, LSPG, LPCL, RPCL | 4.025 | 0.001 |
| *Subcortical* | RCAU | 4.025 | 0.001 |
| **Beta difference:**  **periodic component** |  |  |  |
| *Cortical* | LSFGdor, RSFGdor, LORBsup, RORBsup, LMFG, LORBmid, RORBmid, RIFGoperc, LIFGtriang, RIFGtriang, LSMA, RSMA, ROLF, LSFGmed, RSFGmed, LORBsupmed, RORBsupmed, RREC, LACG, RACG, LDCG, RDCG, LSPG, LPCL, RHES, RITG | 3.394 to 4.025 | 0.003 to 0.001 |
| *Subcortical* | RHIP, RCAU | 4.025 | 0.001 |

*The analysis of gamma-band power in the periodic component revealed a similar picture as in the non-separated data, with significant power increases across a range of cortical and subcortical brain regions,* *highlighting that oscillatory activity is involved in the Ketamine-induced increases of gamma-band power.*

*We repeated these analyses with the simulated data (see following Figure and Table). Interestingly, a strong increase in alpha power and decrease in theta power could be observed in several conditions, contrasting the results seen in the human MEG data. A decrease in beta power could only be observed in conditions with manipulated NMDA-Rs of pyramidal neurons in the non-separated data; in PV and SST conditions an increase in beta power was found. The results of gamma-band power in the periodic signal confirm largely what was observed in the non-separated data, with increases occurring only in PV conditions (PV -60%) and SST conditions.*

|  | **Significant simulation conditions (after FDR correction)** | |
| --- | --- | --- |
|  | **non-separated data** | **periodic component** |
| theta (4-8 Hz) | *decrease:*  all Pyr, VIP -80%, all neurons | *decrease:*  PV -60%, all SST (except -40%), VIP -80% |
| alpha (8-12 Hz) | *increase:*  all PV (except -10%), all SST, VIP -80% | *increase:*  all PV (except -10%),  all SST (except -­40%), VIP -80% |
| beta (13-30 Hz) | *increase:*  all PV (except -10%), all SST  *decrease:*  all Pyr | *increase:*  PV -40%, PV -60% |
| beta (15-30 Hz) | *increase:*  all PV (except -10%), all SST  *decrease:*  all Pyr | - |
| gamma (30-90 Hz) | *increase:*  all PV (except -10%), all SST  *decrease:*  all Pyr, all neurons | *increase:*  PV -60%, all SST  *decrease:*  all Pyr, all neurons |

*A potential explanation for the difference in the power spectral changes in the lower frequencies in human data after ketamine administration vs. simulated data is that the computational layer 2-3 model targeted only E/I-mechanisms (i.e., NMDA-R output of pyramidal neurons and interneurons). While gamma-band changes may be captured by cortical layer 2-3 pyramidal neuron – interneuron mechanisms, other oscillation frequencies might be more reliant on other neurotransmitter systems, such as cholinergic neurotransmission for alpha and beta (Bauer et al., 2012) and theta (Liljenstrom & Hasselmo, 1995**), as well as on additional brain structures, such as hippocampus for theta (Pignatelli et al., 2012), or other cortical layers (e.g. deeper layers for alpha; Bollimunta et al., 2008). We therefore also did not expect these simulations to match all other power spectral features of our human MEG data.*

*We have added these additional findings to the supplementary materials.*

**3) What are the changes to oscillatory event waveforms in the gamma range following ketamine application (in the MEG recordings and in the model simulated MEG)? These are additional features that the authors could check whether the models with NMDA block can capture.**

***Response:*** *We thank the reviewer for this interesting suggestion. Oscillatory event waveforms are an innovative way of looking at oscillatory data and might yield interesting additional insights. For this paper, however, we chose to not investigate this aspect given the already substantial number of analyses conducted in the manuscript.*

**4) Given the significant partial correlation of GRIN2D with aperiodic slope – in what cell types does GRIN2D tend to show higher expression? Also, have any previous experiments demonstrated that GluN2D has enhanced sensitivity to ketamine compared to other NMDA subunits?**

***Response:*** *GRIN2D expression decreases with maturation in many regions, but remains significant in adult hippocampal and cortical interneurons, with minimal expression in principal neurons (Monyer et al. 1994; Thompson et al. 2002; Hanson, et al. 2024). GRIN2D is expressed in about 60–80% of the somatostatin-expressing (SST+) and parvalbumin-expressing (PV+) interneurons in adult mice (Perszyk et al. 2016; von Engelhardt et al 2015).*

*Results on NMDA-R subunit-specific sensitivity to Ketamine have been inconsistent (Zanos et al. 2018), but under physiological conditions, Ketamine may have more pronounced effects on GluN2C and GluN2D receptors compared to GluN2A and GluN2B receptors (Kotermanski & Johnson 2009).*

*We added this discussion to the revised manuscript.*

**5) Can the authors expand their gene expression correlation analysis to include dopaminergic, serotonergic and cholinergic receptor subunits? Similarly, inclusion of other cell type markers (e.g. L1 interneurons or pyramidal cell markers) would equally be informative and interesting.**

***Response:*** *We thank the reviewers for this suggestion. While we agree that these analyses would be interesting and potentially informative, they significantly extend beyond the primary scope of our study, which focuses specifically on E/I-related changes. Incorporating these analyses could lead to a broader exploration better suited for a future study.*

**6) I am not clear on the details of the gene expression vs MEG data correlation analysis (fig 3 and lines 370-381 in the methods). My initial understanding was that each datapoint in the correlation analysis was a brain area (e.g. a centroid in Fig 3B) of mean MEG difference (KET-PLA) vs gene expression. On line 180 describing Fig 3B however it says “Brighter dots indicate higher correlation values”. How can single centroids possess single correlation values? I thought that the brightness represented difference estimates and gene expression values. Please clarify this and add color scales to fig 3B.**

***Response:*** *The brightness of the dots in Figure 3B indeed reflects the difference estimates and gene expression values. We have corrected the figure legend to clarify this, and we have also added a color scale to Figure 3B to better illustrate the data.*

**7) While the simulations showed that both PV and SST could be involved, the gene expression analysis only supported the involvement of PV. So, how much SST is actually involved depends on how much NMDA modulates SST cells to begin with. It’s important to keep in mind that the models (at baseline) assume synapses with equal conductances from NMDA and AMPA so they could be over-estimating the effects of NMDA loss in any particular cell type. As far as I know NMDA activity contributions in human interneurons have not been measured and these models are not fit to any such data. With that in mind, I think this work could benefit from better using the gene expression-MEG correlation results to guide the key simulations to show in the main figures.**

***Response:*** *We agree that a major limitation of the computational model is that it assumes equal conductances for each cell type as well as between NMDA and AMPA receptors. Accordingly, we have added this aspect to the discussion section in the revised manuscript. Regarding the suggestion to use the gene-MEG correlation results to guide the presentation of the simulation figures, we have decided to keep the figures as originally submitted to ensure that all simulated conditions are presented to the readers and that the figure panels are intuitively divided.*

**8) Given the result of PV expression being correlated with larger ketamine-driven effects, it would be relevant to check if increasing the PV cell count in the model increases the effects of NMDA block on simulated MEG.**

***Response:*** *Surprisingly, increasing the PV cell count in the model did not increase the effects of NMDA block on the simulated MEG. We investigated this further and found that, likely due to nonlinear network effects, an increase in PV cell counts resulted in a substantial decrease in PV firing rates leading to a net decrease in PV-mediated inhibition. Thus, the NMDA block resulted in a slight decrease in gamma-band power in this condition.*

**9) I think more conditions in the model could be explored to find realistic contexts where the aperiodic slope is flattened. From the tests that are already shown, decreased NMDA to PV is a good candidate for causing an elevated PSD at higher frequencies (and is supported by the gene expression analysis), and decreased NMDA to Pyr is a good candidate for counteracting the elevated PSD at lower frequencies. Why not explore combinations of just those two targets to see if together they flatten the aperiodic slope?**

***Response:*** *Thanks for this valuable suggestion. We now also analyzed the following combinations of decreased NMDA conductance to PV and Pyr neurons: PV-40% + Pyr-40%, PV-40% + Pyr-20%, PV-60% + Pyr-20%, PV-40% + Pyr-10%. These combinations, however, did not flatten the aperiodic slope either.*

**10) In Mazza et al 2023 reduced inhibition from SST, but not PV, led to a reduced aperiodic slope. Although the mechanism here is slightly different (NMDA input reduction vs inhibitory output reduction), it’s worth discussing why these differences (whether it’s mechanistic-related, or method-related) may be leading to different outcomes.**

***Response:*** *Mazza et al. (2023) reported indeed a flattening of the aperiodic slope in response to reduced SST-mediated inhibition. However, the reduction in inhibition is implemented differently in their work compared to ours. In this highly nonlinear network, these different implementations (reduced activation through reduced NMDA currents vs. reduced output) can potentially lead to differences in the aperiodic slope. There are, however, methodological differences with respect to the calculation of the aperiodic and periodic components of the signal. While we use the IRASA software package, which uses a resampling method to estimate the components, Mazza et al. (2023) use the FOOOF package, which fits Gaussian functions to the signal to estimate the components. Thus, it is hard to determine whether the differences in slope changes between our simulations and the simulations from Mazza et al. (2023) stem from the implementation or the methodological differences.*

**11) Given the focus of this paper on higher frequency oscillations and aperiodic activity, it is unclear if analyzing short simulations will impact the results, but the aperiodic slope is also modulated by lower frequency ranges. Can the authors demonstrate that their 2s simulations generate sufficient power spectra compared to example longer simulations before and after NMDA block?**

***Response:*** *The aperiodic slope was calculated in a frequency range between 7 and 80 Hz, which can be reliably computed by 2s trials. In the human MEG data, we also tested a slope fitting procedure beginning at 3 Hz (see left panel). This, however, did not yield different results but the linear fitting was less accurate. The same occurred when rea-analyzing the slope of the simulation data beginning at 3 Hz (see right panel). We don’t assume that the slope would change considerably for an even lower frequency limit.*

*Also, all simulations were recorded for a length of 3s, of which the first second was removed. This was done to avoid potentially disrupted onset activity. We updated this description in the revised manuscript.*

**12) It’s worth considering some conceptual differences with the model considerations in Yao et al 2023 where depression was simulated via reduced SST inhibitory output conductance. Ketamine is a rapid antidepressant and these results suggest that reducing NMDA drive to SST interneurons may underlie ketamine effects. Thus, a loss of SST interneuron contributions is expected in both depression and when applying a rapid antidepressant. Can the authors discuss this counterintuitive result?**

***Response:*** *As the reviewer correctly pointed out, a reduction in SST interneuron activity is associated with both depression and Ketamine administration, which might seem contradictory at first. In depression, there is a chronic reduction in SST interneuron inhibition, leading to increased baseline cortical activity and impaired signal-to-noise ratio (Duman et al., 2019). This long-term deficit in inhibition results in dysfunctional cortical processing. Ketamine’s antidepressant effects are thought to be mediated through a transient reduction in NMDA receptor activity on SST interneurons. This brief disinhibition triggers a cascade of events that ultimately lead to enhanced synaptic plasticity and improved cortical function (for a review, see Zanos & Gould, 2018).*

**13) While the experiments specifically look at sub-anesthetic administration of ketamine, it is unclear if the simulated NMDA blocks correspond to such a dosage – effects appeared most apparent with PV and SST starting at a 40% reduction and up to 60% and 100%, respectively. Can the authors provide a rationale for why these values represent the action of ketamine during sub-anesthetic dose administration?**

***Response:*** *To our knowledge, no specific values of PV or SST cell block proportions have been reported in former research. Therefore, as we did not have a predefined value for the degree of NMDA receptor reduction that would match a sub-anesthetic ketamine dosage, we tested several different levels of reduction. As the reviewer noted, effects in our data became apparent starting at 20% of PV interneurons and 40% of SST interneurons. While these reductions might seem large, they are not inconsistent with the action of sub-anesthetic ketamine. At sub-anesthetic doses, ketamine likely targets a relatively low proportion of cells, primarily affecting specific cell types (such as interneurons) rather than pyramidal cells. Even with a 40% reduction in NMDA signaling at e.g. PV cells, ketamine would still predominantly impact a subset of cells, and not the majority of neurons in the network.*

**14) The effects of NMDA activity reductions on power spectral metrics in the model are not quantitatively shown. Can the authors show plots linking the mechanistic changes in the model with changes in the power spectral metrics (gamma power and aperiodic slope – like fig 2C & F)? As well, can the authors demonstrate what percent changes in the model lead to similar percent changes in power spectral features seen experimentally during ketamine application?**

***Response:*** *We unfortunately cannot plot the power spectral metrics of every simulation at baseline compared to the same manipulated simulation (connected as in fig 2C &F) because for every single simulation, different random seeds for composing the neuronal population were used.*

*Regarding the second question, we added information on the mean percent changes in power spectral features in the revised manuscript. In the human data, a mean percent change in gamma-band power from Placebo to Ketamine of 21.05% could be observed (PLApost power=0.001819, KETpost power=0.0002202). An increase in approximately the same magnitude could also be observed in the modeled conditions PV-40% (26.16%) and SST-40% (22.09%).*

**15) Fig 6 appears to be taken directly from Yao et al 2022 and Mazza et al 2023. While this is acknowledged in the caption, panel C is a bit misleading given the differences in simulation time, EEG vs MEG methods, and analysis methods. I would suggest replacing panel C with something that illustrates the MEG simulation method that they added (lines 408-414). Adding a panel to better illustrate the key model manipulations would also be helpful. I also find that having this type of figure before presenting the results (or combined with the results figures) generally makes it easier for the reader (otherwise one must skip to the last figure to understand what’s going on).**

***Response:*** *We agree that the previous panel C was misleading, as it did not reflect the data from our study. We therefore followed the reviewer’s suggestion to remove panel C and add an illustration of the model manipulations to panel B. Additionally, we expanded the description of the model in the Results section and thereby now refer to this figure before presenting the results.*

**Minor**

**16) The organization of figs 1 and 2 with the corresponding results text is non-sequential – can the authors re-organize (either the text or the plots) for it to read better (i.e. section 1 -> fig 1, section 2 -> fig 2)?**

***Response:*** *We appreciate the reviewer’s suggestion regarding the organization of Figures 1 and 2. However, we believe that the current structure, where the text is divided into gamma and slope results, is the most intuitive and easier to follow. Figure 2 is designed to show the main results at the virtual channel level for both slope and gamma changes together, providing a comprehensive view of these related outcomes in a single figure. We feel this organization best facilitates the reader's understanding of the key findings.*

**17) Fig 2D looks at the aperiodic component, but this looks like it’s just the averaged raw PSDs being plotted in that frequency range. Can the authors also plot the mean aperiodic fits?** **In Fig S2 it would also be beneficial to show the mean aperiodic fits. Also in Fig S2, for better visibility, can the authors zoom in on the ranges in the 7-30 Hz and 30-80 Hz cases rather than highlighting them on the larger scale (or show the zoomed in versions as insets)?**

***Response:*** *Figure 2D shows, as the legend correctly indicates, the averaged aperiodic component of the PSD. We agree that plotting the mean aperiodic fits is more intuitive for a depiction of aperiodic slope changes and added these. Regarding the depiction of Figure S2, we decided to not zoom in on the respective frequency ranges but keep showing the full range so that it is clear to the reader that the data that was used in the main analysis is only divided into two parts for this additional analysis and no new data is involved.*

**18) Fig S2: It’s interesting that significant ketamine effects on aperiodic slope were only seen in subcortical regions when the whole frequency range was considered – can the authors discuss this result and give more context to it?**

***Response:*** *One possible explanation for this result is that subcortical signals are generally more challenging to detect with MEG due to their weaker strength and greater distance from the sensors. Lower-frequency components of these signals are more likely to survive this attenuation compared to higher frequencies, which could make slope changes more prominent and easier to detect when the entire frequency range is considered. Given these considerations, we are cautious about over-interpreting this finding, as it could reflect methodological limitations rather than a physiological phenomenon.*

**19) It’s not clear why VIP and Pyr neurons were only analyzed for a -80% and -80%/-40% decreases in NMDA whereas PV and SST had multiple levels explored.**

***Response:*** *Our primary focus was on the effects of NMDA reductions in PV+ and SST+ interneurons as these interneuron subtypes were the most relevant for our hypotheses. To ensure the robustness of the observed effects, we explored multiple reduction levels for these cell types. VIP and pyramidal neurons, on the other hand, served more as control conditions to test whether similar effects would emerge. Even with the limited reduction levels analyzed (-80% for VIP and -80%/-40% for pyramidal), we observed that these manipulations did not replicate the patterns seen in the human MEG data. Based on these results, we did not pursue further reduction levels for these cell types, as they did not appear to contribute meaningfully to the observed phenomena.*

**20) In the methods, when detailing NMDA reductions in specific cell types, are the authors referring to NMDA conductance when they say “NMDA activity”? Please clarify this detail.**

***Response:*** *It is correct that the term “NMDA activity” refers to “NMDA conductance”. We agree that the term “conductance” is less ambiguous and therefore changed it in the revised version of the manuscript.*

**21) Lines 388-389: The data provenance descriptions and refs for the Yao et al model are not quite correct. Please consult table S1 in Yao et al 2022, for a full list of data provenance, and correct these details where applicable.**

***Response:*** *Thank you for bringing this to our attention. We have updated the description:*

*“Morphological, electrophysiological and anatomical (density of neurons and synaptic connectivity) features within the microcircuit model were fit to human data where possible and to rodent data otherwise, and show close matches with the experimental characteristics of the modelled populations [74, 76-81].”*

**22) Fig S3 caption appears to have a typo: “PV -40%, SST -20%, PV -15%, pyramidal neurons -5%” I think should say “PV -40%, SST -20%, VIP -15%, pyramidal neurons -5%”.**

***Response:*** *We corrected this typo in the revised text.*

**Reviewer #3**

**The study investigates how ketamine influences the balance of excitation and inhibition in cortical microcircuits, particularly focusing on the roles of parvalbumin and somatostatin interneurons. By analyzing MEG data from 12 participants, the authors explore the changes in gamma-band power and the correlation with gene expression.
Key Findings:
1. Increased Gamma-Band Power: The authors report a significant increase in gamma-band power following Ketamine administration, particularly in prefrontal and central regions.
2. Flatter Aperiodic Slope: The observation of a flatter aperiodic slope is noteworthy, as it suggests increased excitation.
3. Interneuron Dynamics: The computational modeling aspect of the study is a significant contribution, as it attempts to link NMDA receptor dysfunctions in specific interneurons to observed changes in gamma-band power. However, the model's inability to account for changes in the aperiodic slope raises questions about its completeness.**

***Response:*** *We thank the reviewer for their thoughtful feedback on our work. We have revised the manuscript to address the questions raised.*

**1) It is suggested that the authors also include analysis of e.g. delta/theta/alpha/beta and potentially HFO bands to support the aperiodic and gamma-band findings. If gamma-band is sufficient for the main red thread of the paper, then this information could be presented as supplementary information. However, in figure 2D, a clear reduction in 10-20 Hz power is visible suggesting that this frequency range may also be involved in the reduction in the aperiodic slope. How was the balance point set at 30 Hz and would the conclusion have been different had it been set at e.g. 20 or 25 Hz?**

***Response:*** *In the following, the power spectra of both the periodic component and, for comparison, the typical, non-separated fourier-transformed data are shown (hanning-tapered; averaged across all virtual channel regions; the shaded envelopes indicate the standard error).*

*As can be seen, lower frequency ranges, including theta (4-8 Hz) and alpha (8-12 Hz) band, are characterized by high variability, in both non-separated and periodic data. Even though a trend of increased theta power and decreased alpha power can be seen, which is in line with results from other groups (Muthukumaraswamy et al., 2015; De la Salle et al., 2016; Curic et al., 2021) statistical testing at the virtual channel level (FDR-corrected t-tests) revealed no significant theta or alpha power changes in any brain region (p > 0.05).*

*However, significant ketamine-induced decreases in the beta (13-30 Hz) range in several brain regions could be observed, both in the periodic and the non-separated data, confirming our previous findings (Rivolta et al., 2015).*

|  | **Significant brain regions** | **t-values** | **p-values** |
| --- | --- | --- | --- |
| **Beta difference:**  **non-separated data** |  |  |  |
| *Cortical* | LSFGdor, LMFG, LIFGtriang, LSMA, RSMA, LDCG, RDCG, LSPG, LPCL, RPCL | 4.025 | 0.001 |
| *Subcortical* | RCAU | 4.025 | 0.001 |
| **Beta difference:**  **periodic component** |  |  |  |
| *Cortical* | LSFGdor, RSFGdor, LORBsup, RORBsup, LMFG, LORBmid, RORBmid, RIFGoperc, LIFGtriang, RIFGtriang, LSMA, RSMA, ROLF, LSFGmed, RSFGmed, LORBsupmed, RORBsupmed, RREC, LACG, RACG, LDCG, RDCG, LSPG, LPCL, RHES, RITG | 3.394 to 4.025 | 0.003 to 0.001 |
| *Subcortical* | RHIP, RCAU | 4.025 | 0.001 |

*The analysis of gamma-band power in the periodic component revealed a similar picture, with significant power increases across a range of cortical and subcortical brain regions, highlighting that oscillatory activity is involved in the Ketamine-induced increases of gamma-band power.*

|  | **Significant simulation conditions (after FDR correction)** | |
| --- | --- | --- |
|  | **non-separated data** | **periodic component** |
| theta (4-8 Hz) | *decrease:*  all Pyr, VIP -80%, all neurons | *decrease:*  PV -60%, all SST (except -40%), VIP -80% |
| alpha (8-12 Hz) | *increase:*  all PV (except -10%), all SST, VIP -80% | *increase:*  all PV (except -10%),  all SST (except -­40%), VIP -80% |
| beta (13-30 Hz) | *increase:*  all PV (except -10%), all SST  *decrease:*  all Pyr | *increase:*  PV -40%, PV -60% |
| beta (15-30 Hz) | *increase:*  all PV (except -10%), all SST  *decrease:*  all Pyr | - |
| gamma (30-90 Hz) | *increase:*  all PV (except -10%), all SST  *decrease:*  all Pyr, all neurons | *increase:*  PV -60%, all SST  *decrease:*  all Pyr, all neurons |

*A potential explanation for the difference in the power spectral changes in the lower frequencies in human data after ketamine administration vs. simulated data is that the computational layer 2-3 model targeted only E/I-mechanisms (i.e., NMDA-R output of pyramidal neurons and interneurons). While gamma-band changes may be captured by cortical layer 2-3 pyramidal neuron – interneuron mechanisms, other oscillation frequencies might be more reliant on other neurotransmitter systems, such as cholinergic neurotransmission for alpha and beta (Bauer et al., 2012) and theta (Liljenstrom & Hasselmo, 1995), as well as on additional brain structures, such as hippocampus for theta (Pignatelli et al., 2012), or other cortical layers (e.g. deeper layers for alpha; Bollimunta et al., 2008). We therefore also did not expect these simulations to match all other power spectral features of our human MEG data. We have added these additional findings to the supplementary materials.*

*Referring to the reviewer’s last question, we computed the slope between 7 and 20 Hz and found no significant slope differences either.*

**2) The “disinhibition hypothesis” or E/I balance is controversial and has been challenged from many sides. It may be advantageous to take this in, as the computational model does not account for the changes in slope seen in the MEG-data. Furthermore, in rodent experiments in awake and freely moving animals both ketamine and PCP induced increased firing from pyramidal cells without reduction in the activity of local inhibitory interneurons. This further challenges the traditional "disinhibition hypothesis" that suggests NMDA receptor antagonists primarily work by inhibiting GABAergic interneurons to enhance pyramidal neuron activity.**

***Response:*** *We thank the reviewer for these comments. We incorporated additional discussion in the revised manuscript regarding the challenges to the disinhibition hypothesis.*

*We appreciate the mentioning of the Amat-Foraster et al. (2019) study with the finding of no decrease in local interneuron activity after Ketamine administration. We believe that our results show that this study does not necessarily contradict the disinhibition hypothesis. As can be seen in Figure 6 (previously Figure 5), when only NMDA-Rs of a specific interneuron type (e.g. SST+) are affected, the spike rate of the other interneuron types, such as PV+, increase alongside the spike rates of pyramidal neurons. Amat-Foraster et al. did not differentiate between interneuron subtypes, and it is likely that their analysis grouped predominantly fast-spiking PV+ interneurons into the interneuron category. If ketamine acts primarily on SST interneurons, or on both SST and PV interneurons, it would be conceivable—if not likely—that the spike rates of PV interneurons still increase while the main mechanism for increased pyramidal cell activity (and gamma-band power) is via disinhibition.*

**3) Correlation with gene expression profiles. It would strengthen the paper if the authors also showed correlations to other frequency bands that are affected by ketamine – or that there are none because the gamma band is the more relevant one.**

***Response:*** *We thank the reviewer for this suggestion. However, the primary focus of our study was on gamma-band activity and aperiodic slope changes, as these are well-established measures related to E/I balance. By concentrating on these metrics, we aimed to provide a more targeted and in-depth investigation into the mechanisms underlying ketamine’s effects. While correlations with other frequency bands could be of interest, they fall outside the scope of this study and may be better addressed in future analyses.*

***References***

*Ahmad, J., Ellis, C., Leech, R., Voytek, B., Garces, P., Jones, E., ... & McAlonan, G. (2022). From mechanisms to markers: novel noninvasive EEG proxy markers of the neural excitation and inhibition system in humans. Translational Psychiatry, 12(1), 467.*

*Amat-Foraster, M., Celada, P., Richter, U., Jensen, A. A., Plath, N., Artigas, F., & Herrik, K. F. (2019). Modulation of thalamo-cortical activity by the NMDA receptor antagonists ketamine and phencyclidine in the awake freely-moving rat. Neuropharmacology, 158, 107745.*

*Bauer, M., Kluge, C., Bach, D., Bradbury, D., Heinze, H. J., Dolan, R. J., & Driver, J. (2012). Cholinergic enhancement of visual attention and neural oscillations in the human brain. Current Biology, 22(5), 397-402.*

*Bollimunta, A., Chen, Y., Schroeder, C. E., & Ding, M. (2008). Neuronal mechanisms of cortical alpha oscillations in awake-behaving macaques. Journal of Neuroscience, 28(40), 9976-9988.*

*Curic, S., Andreou, C., Nolte, G., Steinmann, S., Thiebes, S., Polomac, N., ... & Mulert, C. (2021). Ketamine alters functional gamma and theta resting-state connectivity in healthy humans: Implications for schizophrenia treatment targeting the glutamate system. Frontiers in psychiatry, 12, 671007.*

*De la Salle, S., Choueiry, J., Shah, D., Bowers, H., McIntosh, J., Ilivitsky, V., & Knott, V. (2016). Effects of ketamine on resting-state EEG activity and their relationship to perceptual/dissociative symptoms in healthy humans. Frontiers in Pharmacology, 7, 348.*

*Duman, R. S., Sanacora, G., & Krystal, J. H. (2019). Altered connectivity in depression: GABA and glutamate neurotransmitter deficits and reversal by novel treatments. Neuron, 102(1), 75-90.*

*von Engelhardt, J. et al. GluN2D-containing NMDA receptors-mediate synaptic currents in hippocampal interneurons and pyramidal cells in juvenile mice. Front Cell Neurosci 9 (2015).*

*Hanson, J. E. et al. Therapeutic potential of N-methyl-D-aspartate receptor modulators in psychiatry. Neuropsychopharmacol. 49, 51–66 (2024).*

*Kotermanski, S. E., & Johnson, J. W. (2009). Mg2+ imparts NMDA receptor subtype selectivity to the Alzheimer's drug memantine. Journal of Neuroscience, 29(9), 2774-2779.*

*Liljenstrom, H. A. N. S., & Hasselmo, M. E. (1995). Cholinergic modulation of cortical oscillatory dynamics. Journal of Neurophysiology, 74(1), 288-297.*

*Mazza, F., Guet-McCreight, A., Valiante, T. A., Griffiths, J. D., & Hay, E. (2023). In-silico EEG biomarkers of reduced inhibition in human cortical microcircuits in depression. PLoS Computational Biology, 19(4), e1010986.*

*Monyer, H., Burnashev, N., Laurie, D. J., Sakmann, B. & Seeburg, P. H. Developmental and regional expression in the rat brain and functional properties of four NMDA receptors. Neuron 12 , 529–540(1994).*

*Muthukumaraswamy, S. D., Shaw, A. D., Jackson, L. E., Hall, J., Moran, R., & Saxena, N. (2015). Evidence that subanesthetic doses of ketamine cause sustained disruptions of NMDA and AMPA-mediated frontoparietal connectivity in humans. Journal of Neuroscience, 35(33), 11694-11706.*

*Perszyk, R. E. et al. GluN2D-Containing N-methyl-d-Aspartate Receptors Mediate Synaptic Transmission in Hippocampal Interneurons and Regulate Interneuron Activity. Mol. Pharmacol. 90, 689–702 (2016).*

*Pignatelli, M., Beyeler, A., & Leinekugel, X. (2012). Neural circuits underlying the generation of theta oscillations. Journal of Physiology-Paris, 106(3-4), 81-92.*

*Rivolta, D., Heidegger, T., Scheller, B., Sauer, A., Schaum, M., Birkner, K., ... & Uhlhaas, P. J. (2015). Ketamine dysregulates the amplitude and connectivity of high-frequency oscillations in cortical–subcortical networks in humans: evidence from resting-state magnetoencephalography-recordings. Schizophrenia bulletin, 41(5), 1105-1114.*

*Thompson, C. L., Drewery, D. L., Atkins, H. D., Stephenson, F. A. & Chazot, P. L. Immunohistochemical localization of N-methyl-D-aspartate receptor subunits in the adult murine hippocampal formation: evidence for a unique role of the NR2D subunit. Brain Res Mol Brain Res 102, 55–61 (2002).*

*Zanos, P., Moaddel, R., Morris, P. J., Riggs, L. M., Highland, J. N., Georgiou, P., ... & Witkin, J. M. (2018). Ketamine and ketamine metabolite pharmacology: insights into therapeutic mechanisms. Pharmacological reviews, 70(3), 621-660.*

*Zanos, P., & Gould, T. D. (2018). Mechanisms of ketamine action as an antidepressant. Molecular psychiatry, 23(4), 801-811.*
