# Supplementary material for: Computational modeling of ketamine-induced changes in gamma-band oscillations: The contribution of parvalbumin and somatostatin interneurons
Source: PLoS Comput Biol. 2025 Jun 9;21(6):e1013118. doi: 10.1371/journal.pcbi.1013118 (PMC12204622; doi:10.1371/journal.pcbi.1013118)
Supplement: Attachment — Submitted filename: Response to Reviewers[1].docx [file pcbi.1013118.s009.docx]

**Response to Reviewers**

**Reviewer #2:**

**Reviewer’s response to point 1:** **In their response the authors try to make the case that the two measures are not measures of the same effect. However, the points that they give only seem to argue for them being driven by a similar effect. For example, they mentioned that there is a strong correlation between aperiodic slope and gamma power. They say that, albeit not a perfect relationship, it is not possible to disentangle the effect and show that gamma power is not driven by aperiodic slope. This only convinces me further that the two metrics are likely two measurements of the same effect. I’d suggest that this should be addressed more clearly in their manuscript, even if just as a caveat.**

***Response****: We thank the reviewer for highlighting this important point. We have now addressed this caveat in the manuscript:*

*“**While gamma-band power and aperiodic slope were used as distinct markers of E/I-balance, these measures are strongly correlated. Therefore, it cannot be ruled out that changes in gamma-band power are driving the change in aperiodic slope in our MEG data, and that they might reflect overlapping aspects of the same underlying mechanism. “*

**The correlation might actually be due to a peak on the upper end of the frequency range – does stopping at 80/90 Hz cut into gamma peaks, such that gamma peaks end up affecting the flatness of the aperiodic component? In fact, the upward inflection in the right tail of the separated periodic component (from the point 2 checks) suggests that there is a peak being cut near 80-90 Hz. Does extending the range past this peak (or even decreasing to before the peak) change the findings on aperiodic slope? And if not, is the correlation between aperiodic slope and gamma amplitude still present?**

***Response****: We repeated the analyses with the slope fitted between 7 and 70 Hz and between 7 and 100 Hz. As can be seen in the Figure below, the results did not differ from the slope fitted between 7 and 80 Hz, the peak at around 80-90 Hz therefore does not seem to have an effect. Moreover, the correlation between aperiodic slope and gamma amplitude was still very high when fitted between 7 and 70 Hz (rho = -.87; p < .001) and between 7 and 100 Hz (rho = -.80; p = .002).*

**Reviewer’s response to point 7:** **Regarding my point on using the gene expression-MEG analysis to guide simulations - if it was expected that SST and VIP expression contribute to ketamine-driven changes in gamma and aperiodic slope, please discuss possible reasons why they were not correlated with these changes. That is, discuss whether there are methodological issues that might occlude these correlations or if these are likely true negative results. Although the authors provide some justifications (in the intro and discussion) for simulating NMDA changes to SST and VIP interneurons, because these were not supported by the expression-MEG correlation results, the simulations come across as being disjointed from the gene expression-MEG correlation results.**

***Response****: We agree that more discussion on the gene expression-MEG correlations, particularly the non-significant findings for SST expression, helps to better integrate these results with the outcomes of the simulations. Although not significant, we did identify partial correlations of SST expression with gamma and especially with slope changes of considerable size (.28 and .36, respectively). These correlations, however, did not reach significance when correcting for spatial autocorrelation.* *Together with the simulation results showing that NMDA-R malfunction in SST interneurons reproduces increased gamma power as in human MEG data, we assume that these correlations might not be true negative results but rather supporting the idea that SST-related effects may exist but are more subtle than those associated with PV expression. We have now added this interpretation to the manuscript:*

*“The correlation of SST gene expression with changes in gamma-band power and aperiodic slope was also of considerable size, but did not reach statistical significance after correcting for spatial autocorrelation. Together with the simulation results showing that NMDA-R malfunction in SST+ interneurons reproduces increased gamma power as in human MEG data, these findings might indicate that SST-related effects may exist but are more subtle than those associated with PV gene expression.”*

*The lack of correlation between VIP gene expression and gamma/slope measures was, on the other hand, not surprising. Given the disinhibiting role of VIP+ interneurons in E/I-balance and the fact that most studies have not identified a contribution of VIP+ interneurons towards the effects of Ketamine, we did not expect a significant correlation. This finding is also consistent with our simulation results.*
